# Mechanosensitive Piezo1 protein as a novel regulator in macrophages and macrophage-mediated inflammatory diseases

**DOI:** 10.3389/fimmu.2023.1149336

**Published:** 2023-06-02

**Authors:** Yu Tang, Chuanxiang Zhao, Ying Zhuang, Anjing Zhong, Ming Wang, Wei Zhang, Liqun Zhu

**Affiliations:** ^1^ Department of Pathology, Affiliated Hospital of Jiangsu University, Zhenjiang, Jiangsu, China; ^2^ Institute of Medical Genetics and Reproductive Immunity, School of Medical Science and Laboratory Medicine, Jiangsu College of Nursing, Huai’an, Jiangsu, China; ^3^ Department of Medical Imaging, Affiliated Hospital of Jiangsu University, Zhenjiang, Jiangsu, China; ^4^ Department of Gastroenterology, Affiliated Hospital of Jiangsu University, Zhenjiang, Jiangsu, China

**Keywords:** Piezo1 channel, macrophages, inflammatory diseases, mechanical forces, mechanotransduction

## Abstract

Macrophages are the most important innate immune cells in humans. They are almost ubiquitous in peripheral tissues with a large variety of different mechanical milieus. Therefore, it is not inconceivable that mechanical stimuli have effects on macrophages. Emerging as key molecular detectors of mechanical stress, the function of Piezo channels in macrophages is becoming attractive. In this review, we addressed the architecture, activation mechanisms, biological functions, and pharmacological regulation of the Piezo1 channel and review the research advancements in functions of Piezo1 channels in macrophages and macrophage-mediated inflammatory diseases as well as the potential mechanisms involved.

## Introduction

Macrophages are crucial cells in the innate immune system, contributing to maintenance of tissue development and homeostasis, clearance host defense during pathogen infection, and promotion of tissue repair in response to tissue injury (1). They reside in specific tissues, such as the heart, lungs, liver, intestines, and skin. Studies have revealed that tissue-resident macrophages are adaptable to the local functional needs, mechanical stimulation, and regulation of various mechanical stresses from the extracellular milieu, including substrate rigidity, interstitial flow, stretch, shear force, and elasticity (2). Mechanical stress is converted into electrical, chemical, or biochemical responses that activate intracellular signaling pathways and modulate gene expression to serve cellular function (3). For instance, substrate rigidity could induce macrophages to differentiate into various canonical phenotypes, pro-inflammatory (M1) or anti-inflammatory (4, 5). In addition, substrate rigidity determines the magnitude of frontal-towing force *via* the RhoA kinase ROCK, myosin II, and PI3 kinase, thus precipitating macrophage motility (6).

With respect to the mechanosensors, recent reviews have documented that numerous cell adhesion molecules, such as integrin, selectin, and cadherin, can mediate the cell–cell or cell–matrix interactions and perceive various mechanical stimuli, thus inciting a series of cell responses (7). Another type of mechanosensors consist of mechanosensitive ion channels (MSCs), such as TRP channels, Ca^2+^ channels, K^+^ channels, the hyperosmolality-gated calcium-permeable channels (OSCA) protein family, and the DEG/ENaC superfamily, which allow for the passage of cations including Ca^2+^, Na^+^, and K^+^ along the electrochemical gradient independent of ATP hydrolysis (8). The Piezo channel, which is a novel type of MSC identified in mouse neuroblastoma cell line Neuro2A by Bertrand Coste and colleagues in 2010 (9), contains Piezo1 and Piezo2 proteins with similar structures encoded by Fam38a and Fam38b genes, respectively (10). Piezo1 is highly expressed in the lungs, skin, bladder, kidneys, endothelial cells (ECs), and erythrocytes (9), with a vital role to play in regulation of blood pressure and myoblast fusion during skeletal muscle formation (11, 12). In parallel, Piezo2, which is predominantly expressed in sensory trigeminal ganglia, dorsal root ganglia, Merkel cells, lungs, and bladder (9, 13), serves a critical role in regulation of sensations as mild tactility, proprioception, and bladder distension (14, 15).

Emerging evidence suggests that piezo1 plays an indispensable role in mechanotransduction in macrophages. In this review, we discuss the architecture, activation mechanisms, biological functions, and pharmacological regulation of the Piezo1 channel and summarize the research advances on the Piezo1 channel in macrophages as well as innate immunity and address the potential mechanisms involved.

## Architecture, activation mechanisms, and pharmacological regulation of Piezo1 channel

### Architecture of Piezo1 channel

**Figure 1:**
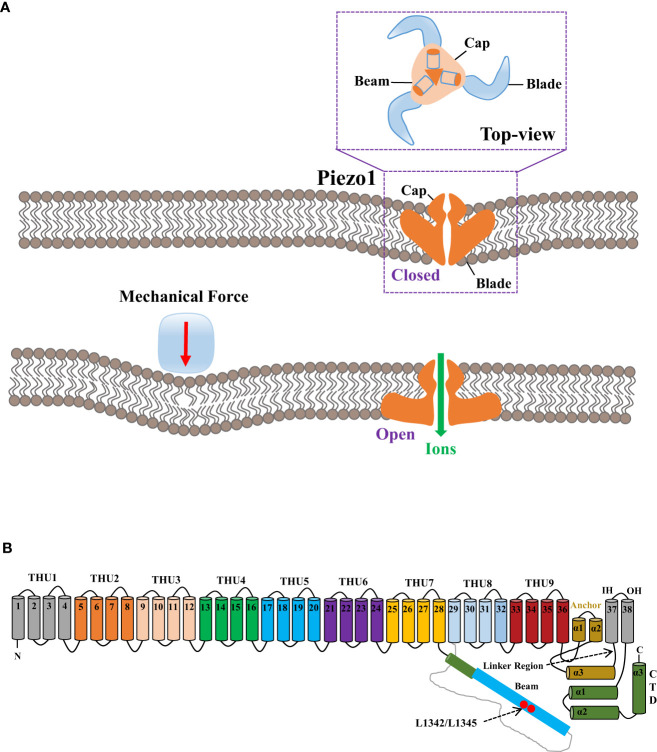
The mechanogating mechanisms by the extracellular force and the 38-TM structure of the mouse Piezo1 channel. **(A)** The mechanogating mechanisms of Piezo1 by the extracellular force. **(B)** Each blade-like propeller is evenly distributed around the central pore and contains unique 38 transmembrane α-helices, as divided into the following structures: N-terminal blade, C-terminal pore region, the long intracellular beam, and anchor domain. The final two α-helices (37 and 38) at the C-terminal, termed as the inner helix and outer helix, respectively, form a central ion pore region of Piezo1 with the C-terminal intracellular domain. The loci of L1342 and L1345 in the long beam structure act as the fulcrum to form an intramolecular lever–like transmission device, thus effectively transmitting and amplifying the subtle mechanical stress or small molecular compounds sensed by transmembrane helical units to the central pore.

Human Piezo1 gene Fam38a is located on chromosome 16, and the mouse Piezo1 gene is highly homologous to humans. Human and mouse Piezo1 protein encompasses 2,521 and 2,547 amino acids, respectively (9, 16). Piezo1 protein is documented as the largest transmembrane molecule with 24–40 transmembrane domains, each being evolutionarily conservative and composed of four groups of repeated transmembrane units (9, 17). Researchers have revealed the structure of the mouse Piezo1 channel by means of protein engineering, X-ray crystallography, single-particle frozen electron microscopy, and live-cell immunostaining (10, 16, 18). From the perspective of three-dimensional architecture, mouse Piezo1 constitutes a trimer in the shape of a three-blade propeller with a central pore domain and three peripheral blade-like propellers (16, 17). The three leafy domains extend outward in the lipid bilayer and constitute a signature nano-bowl structure on the surface of cell membrane (17) (**Figure 1A**). Each blade-like propeller evenly distributed around the central pore contains unique 38 transmembrane α-helices, consisting of the following structures: N-terminal blade, C-terminal pore region, long intracellular beam, and anchor domain (9) (**Figure 1B**). The N-terminal (transmembrane α-helices 1-36) comprises nine repetitive fold structures with four α-helices, termed as transmembrane helical units (THUs) or Piezo repeat, acting as the skeleton of each blade (19). The remaining two α-helices (37 and 38) at the C-terminal, termed as the inner helix (IH) and outer helix (OH), respectively, constitute a central ion pore region of piezo1 with the C-terminal intracellular domain (20). On the top of central ion pore, there is a cap with negatively charged residues composed of the C-terminal extracellular domain (16). The deletion of the area where the cap domain contacts the blade-like propeller would preclude the mechanical activation, indicating the governance of central pore channel by the cap (21, 22). In addition, the anchor domain consists of three helices (α1, α2, and α3) and acts as a bridge between THU9 and the OH-IH pair (22). On the intracellular surface, the long beam is organized at a 30° angle relative to the membrane plane (8). Functionally, the long beam not only supports the blade-like propeller but also physically bridges the THU7-8 loop to the central pore, which renders an ideal structure for mechanical transmission (18).

### The activation mechanisms of Piezo1 channel

There are two models of mechanical perception of the Piezo1 channel including the force-from-lipid (FFL) bilayer model and force-from-filament (FFF) or tether model, which correspond to two mechanical signal transduction directions: inwardly and outwardly, respectively (23, 24). Therefore, factors affecting Piezo1 mechanogating involves cell membrane tension, hardness, cell membrane skeleton protein, and other channels or proteins interacting with Piezo1.

When external mechanical pressure is exerted to the cell membrane, modification of membrane tension could lead to the deformation of the Piezo1 channel structure, changed from curved to flat, thereby resulting in the patency of the central ion pore (18), which could be explicated by the lever-like mechanism hypothesis (25). The blade-like propeller is composed of nine THUs in the Piezo1 channel and serves as the mechanical force sensor, while the loci L1342 and L1345 in its long beam structure act as the fulcrum to form an intramolecular lever–like transmission device, thus effectively transmitting and amplifying the subtle mechanical stress or tiny molecular compounds sensed by THUs to the central pore. Eventually, effective control of the overtness of the Piezo1 channel and selective cation permeability are thus established (25).

However, FFL is applicable to the Piezo1 channel for constant surveillance of variations of local membrane tension and curvature. Due to the restriction of cytoskeleton, membrane tension could only spread locally on the cell membrane (23). The Piezo1 channel could be activated not only by local mechanical stimulation but also by remote mechanical stimulation at the whole cell level (23). As per a recent report, the cap domain of Piezo1 interacts directly with the extracellular domain of E-cadherin while the intracellular gating element of Piezo1 might interact with the E-cadherin cytosolic tail (26). Accordingly, the Piezo1 channel establishes a biochemical and functional connection with the F-actin cytoskeleton *via* the cadherin-β-catenin mechanical transduction complex, effectively rendering the long-distance mechanical conduction across a cell or between cells *via* the cytoskeleton (26).

### Pharmacological regulation of Piezo1 channel

With the extensive research of the Piezo1 channel, some small-molecule drugs have been developed. Chemical agonists of Piezo1, including Yoda1 and Jedi1/2, could activate the Piezo1 ion channel in the absence of mechanical stimulation. Yodal, which contains two chlorine radicals and one sulfide radical, is essential to unlock Piezo1 (27). At micromolar concentrations, Yoda1 acts as a molecular wedge and is directly bound to Piezo1 in the absence of other proteins, facilitating stress-induced conformational changes, effectively reducing the mechanical threshold of the Piezo1 channel for activation and significantly retarding inactivation (28, 29). These findings validate the presence of natural agonists that can unlock Piezo1 independent of any mechanical stimulation. Dooku1, a synthesized analog of Yoda1, was reported to reversibly antagonize Yoda1-induced activation of Piezo1 *via* competition for specific channel binding sites (30). In addition, Jedi1/2, two low-affinity water-soluble chemical activators, could specifically activate Piezo1 with quicker explosion and shorter decay than the Yodal-mediated current (25), wherein Jedi1 and Yoda1 synergistically activate different Piezo1 sites (25).

Piezo1 inhibitors have also been identified, such as ruthenium red (RuR), gadolinium (Gd^3+^), and the GsMTx4 peptide. RuR and Gd^3+^, broad-spectrum mechanical stress-sensitive ion channel inhibitors, can reportedly block mouse Piezo1 with IC50 5.4 μM in the case of extracellular administration (10). The inhibitory effect of RuR on Piezo1 is related to voltage, indicating its dependence on the direction of ion flow (31). GsMTx4, a peptide toxin extracted from spider venom, can reportedly inhibit Piezo1 at low micromolar concentrations (32). However, GsMTx4 might not directly bind Piezo1 unless the midpoint of activation is shifted to a higher-pressure value *via* modulation of local membrane tension near the channel (32–34). Unfortunately, these inhibitors are not Piezo-specific, or rather, they are inhibitors of a number of other ion channels, such as transient receptor potential (TRP) channels (35). Therefore, Piezo1-specific inhibitors still await further exploration.

## Role of Piezo1 channel in macrophages

A wealth of evidence has shown that Piezo1 mRNA, rather than Piezo2, is highly expressed in bone-marrow derived macrophages (36–38). After stimulation by IFN-γ and LPS or “stiff,” macrophages exhibit increased Piezo1 expression (38), suggesting that Piezo1 acts as an important regulator of macrophage function and polarization.

Piezo1 has been shown to regulate macrophage function. According to a study, LPS or the Piezo1 agonist Yodal can incite the interaction between Pizeo1 and TLR4. Moreover, LPS can stimulate TLR4 to induce Ca^2+^ influx requiring Piezo1, thus shedding light on the role of Piezo1 in TLR4 signaling (36, 38). In addition, mice with a Lyz2-Cre-mediated knockout of Piezo1 exhibit no abnormality in the frequency of neutrophils and macrophages in the bone marrow, spleen, and blood. However, Piezo1 deficiency impaired phagocytosis, mitochondrion–phagosomal ROS production, and efficient bacterial clearance of bacteria (36). It can be inferred that Piezo1 is a key regulator of macrophage phagocytic activity (39). Mechanically, Piezo1 regulates the bactericidal activity of macrophages through Ca^2+^ signal–induced activation of the CaMK II-Mst1/2-Rac1-cytoskeleton rearrangement axis (36). To the contrary, CD11b^+^ myeloid cells lacking Piezo1 present decreased proinflammatory cytokines, enhanced peritoneal bacterial clearance, and increased survival in a mouse model of polymicrobial sepsis *via* cecal ligation and puncture (40). Some sporadic studies have demonstrated Piezo1 as a sensor of cyclical hydrostatic pressure (CHP) (37) or “stiffer” (38) in myeloid cells. After stimulation by CHP, macrophage Piezo1 mediates Ca^2+^ influx, resulting in activation of activator protein-1 (AP-1) that drives transcription of endothelin-1 (Edn1). EDN1 signaling, in turn, stabilizes hypoxia-inducible factor 1α (HIF1α) to facilitate the prolonged proinflammatory expression profile, such as Interleukin 1b (IL-1b), Tumor Necrosis Factor alpha (TNF-α), C-X-C motif chemokine ligand 10 (CXCL10), and prostaglandin E2 (37), eventually exacerbating a model of pulmonary inflammation. In summary, Piezo1-mediated-Ca^2+^ influx and the subsequent Ca^2+^ signaling contribute to macrophage function.

Piezo1 has been shown to affect macrophage polarization. Macrophages lacking Piezo1 present with the downregulation of “M1-like” inflammatory marker iNOS with secreting significantly less TNF-α and IL-6 by decreasing NF-κB in response to IFN-γ/LPS stimulation and the upregulation of “M2-like” pro-healing markers Arg1 by enhancing STAT6 activation after IL-4/IL13 treatment (38). The above effect is driven by Piezo1-mediated Ca^2+^ influx and is enhanced by increased substrate rigidity or “stiffness” (38). Ca^2+^ is known to regulate the activation of transcription factors, where increased intracellular Ca^2+^ has been shown to enhance NF-κB and repress STAT6 activation (41, 42). In addition to soluble inflammatory stimuli, stiffness-mediated macrophage activation is dependent on Piezo1 (38). Both static and cyclic stretch can upregulate the integrin CD11b (αM integrin) expression and downregulate Piezo1 expression (43), with a crosstalk in between, since CD11b knockdown upregulates Piezo1 expression; conversely, Piezo1 knockdown upregulates CD11b expression (43). Moreover, knockdown of either CD11b or Piezo1 *via* siRNA-abrogated stretch-mediated changes in inflammatory responses (43). There is a study that three-dimensionally printed Ti2448 with low stiffness enhances angiogenesis and osteogenesis by promoting Piezo1/YAP signaling axis–mediated polarization of macrophages toward the M2 phenotype and related cytokine secretion (44).Therefore, subcutaneous implantation of some biomaterials by surgery is a powerful clinical strategy, with deletion of Piezo1 in macrophages decreasing inflammatory activation and increasing wound healing response.

## The role of Piezo1 channel in inflammatory diseases

**Figure 2:**
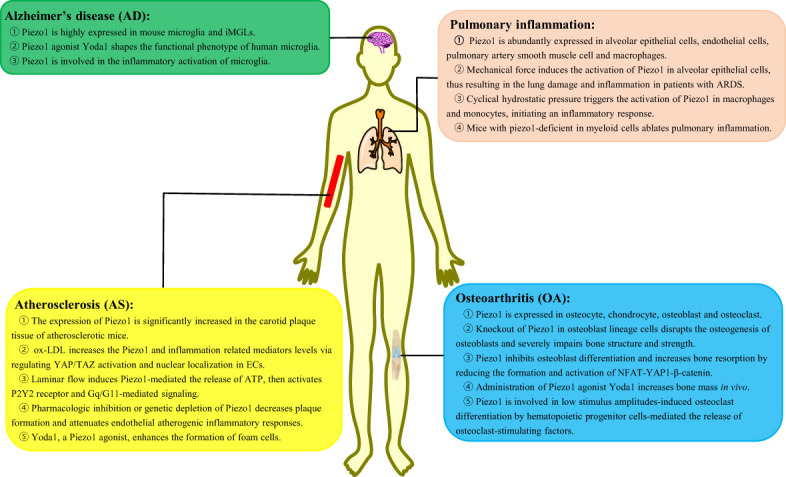
The role of Piezo1 in macrophage-mediated inflammatory diseases. Piezo1 regulates the inflammatory responses, such as Alzheimer’s disease, pulmonary inflammation, atherosclerosis, and osteoarthritis. iMGLs, human-induced pluripotent stem cell–derived microglia-like cells; ARDS, acute respiratory distress syndrome; ox-LDL, oxidized low-density lipoprotein; ECs, endothelial cells.

Inflammation is an immune defensive mechanism in response to pathogen infection or tissue injury to eliminate pathogens or promote damaged tissue restoration [45]. Inflammation is accompanied by leukocytosis, pain, heat, redness, swelling, and organ dysfunction (45). Macrophages are known as phagocytic innate immune cells that stem from the myeloid lineage and differentiated from monocytes in tissues. Macrophages are present in most tissues such as microglia in the central nervous system, alveolar macrophages in the lungs, Kupffer cells in the liver, and osteoclasts in the bone (46). Macrophages play a critical role in the initiation, maintenance, and resolution of inflammation. This section focuses on the in-depth investigation of the role of Piezo1 in macrophage-mediated inflammatory diseases, such as Alzheimer’s disease (AD), pulmonary inflammation, atherosclerosis, and osteoarthritis (OA) (**Figure 2**).

### Alzheimer’s disease

AD is a progressive neurodegenerative disease characterized by proliferation and activation of microglia, accumulated around amyloid-β (Aβ) plaques (47). There is mounting evidence pointed to a crucial role for microglia in the pathogenesis of AD, the mechanism of which is the inability of microglia to clear accumulating Aβ plaques and their proinflammatory functions (48). Under normal conditions, microglia could migrate to the pathological sites, with clearance of damaged tissue and Aβ plaques *via* phagocytosis (49). In the brain, deposited Aβ plaques are rather brittle and hard and their stiffness is approximately 3 × 10^9^ Pa (50). Thus, microglia are attracted to stiff regions *in vitro* and activated by implantation of stiff foreign bodies *in vivo*, implying the presence of specific mechanosensors in microglia (51, 52).

Recently, a study has reported the high expression of Piezo1 on the cellular membrane and nucleus both in mouse microglia and human-induced pluripotent stem cell–derived microglia-like cells (53). When Piezo1 is activated by the agonist Yodal, the functional phenotype of human microglia is shaped, such as enhanced migration, phagocytosis, and lysosomal activity, thus orchestrating Aβ clearance *in vivo* (53). Intriguingly, Aβ suppressed Piezo1 activity *via* an unknown mechanism in microglia that might be attributed to an Aβ self-protection mechanism (53). However, another report has revealed that the activation of Piezo1 decreases the microglial proliferation and cell viability as well as inhibits the migration ability of microglia in an acute high-glucose stress (54). In addition to the ability of microglia to clear Aβ plaques, Piezo1 is involved in the inflammatory activation of microglia. Lipopolysaccharide (LPS) upregulates the expression of Piezo1 in microglia, and Piezo1 upregulation significantly attenuates LPS-induced expression of proinflammatory cytokines TNFα, IL-1β, and IL-6 *via* the JNK1 and mTOR signaling pathways (54).

In conclusion, the studies have demonstrated the abundance and functional expression of Piezo1 channels in microglia. Whether Piezo1 may become a new drug target for AD will be the focus of future research.

### Pulmonary inflammation

Pulmonary inflammation is a common disease caused by infection, physical and chemical factors, immune injury, allergy, and drugs. Lung tissue is a highly mechanized organ and is exposed to complex pulmonary hemodynamics and respiratory mechanics. Cells in the lungs, such as macrophages, bear mechanical stress (55). There is plenty of evidence that mechanical stress plays a critical role in pulmonary inflammation (56).

It is well acknowledged that Piezo1 is abundantly expressed in alveolar epithelial cells (57), ECs (58), smooth muscle cells of the pulmonary artery (59), and macrophages (37). Piezo1 responds to lung mechanical stress and is involved in the development of lung inflammation by multiple mechanisms. Mechanical stress induces the activation of Piezo1 in alveolar epithelial cells, resulting in the increase of intracellular Ca^2+^ and inducing cell apoptosis and abnormal secretion of alveolar surfactants (60), thereby aggravating lung damage and inflammation in patients with acute respiratory distress syndrome (61). Additionally, there are also reports unveiling that CHP in the lungs triggers the activation of Piezo1 in macrophages and monocytes and initiates an inflammatory response (37). Piezo1 enhances the secretion of CXCL2 in monocytes, thus allowing neutrophils to migrate from the blood to the lungs and evoking neutrophils to clear bacteria (62). Mice with Piezo1 deficiency in myeloid cells reportedly present ablated pulmonary inflammation by decreasing numbers of lung-infiltrating neutrophils and levels of inflammatory mediators in *Pseudomonas aeruginosa* infection or fibrotic autoinflammation (37).

Based on the important roles Piezo1 plays in lung inflammation, Piezo1 is expected to be a potential therapeutic target for pulmonary inflammation.

### Atherosclerosis

Atherosclerosis (AS) is a chronic inflammation disease resulting from various risk factors, such as obesity, hypertension, diabetes, stroke, inflammatory mediators, and high plasma levels of LDL cholesterol and triglycerides (63). AS is initiated by injury of vascular ECs, which is caused by a number of factors, including mechanical forces such as shear stress and stretching of blood flow (64). Subsequently, monocytes in the blood are recruited into the endothelium and differentiate into macrophages by the deposited cholesterol and lipids; afterward, macrophages phagocytize lipids to transform into foam cells, which accumulate to form lipid stripes and lipid plaques as well as secrete proinflammatory factors (65). Studies show that the expression of Piezo1 was significantly increased in the carotid plaque tissue of atherosclerotic mice (66). Accumulating evidence suggests various functions of Piezo1 involvement in AS.

Piezo1 regulates ECs and macrophages of different cellular processes in AS. A study found that Piezo1 was abundantly expressed in ECs (67). Oxidized low-density lipoprotein (ox-LDL) can increase the Piezo1 expression and inflammation-related mediators (JNK, TNF-α, and NF-κB) *via* regulation of YAP/TAZ activation and nuclear localization in ECs (66). Furthermore, turbulent shear of blood flow triggers the activation of Piezo1, inducing inflammatory signaling *via* integrin-associated PECAM-1/VE-calmodulin/VEGFR2 and PI3-kinase-dependent activation, further leading to FAK-dependent NF-kB activation (68, 69). Laminar flow induces Piezo1-mediated release of ATP and then activates the P2Y2 receptor and Gq/G11-mediated signaling, which further leads to the phosphorylation of AKT and the release of eNOS in antiatherosclerosis involvement (11, 68). As expected, pharmacologic inhibition or genetic depletion of Piezo1 decreases plaque formation and attenuates endothelial atherogenic inflammatory responses (66–68). Furthermore, Piezo1 is also evidenced to be expressed in both THP-1 and RAW264.7 cells (70). Atherosclerotic plaque–induced blood flow shear stress can promote the monocyte activation *via* Piezo1, enhancing phagocytic activity and LDL uptake and cytokine expression (71). Yoda1, a Piezo1 channel agonist, enhanced the formation of foam cells, which is inhibited by AS treatment drug salvianolic acid B (SalB) (67). Transcatheter aortic valve implantation (TAVI) has been known as an effective treatment for aortic valve stenosis. Studies reveal that TAVI represents an anti-inflammatory regimen by reduction of Piezo1-mediated monocyte activation (71).

In brief, Piezo1 is a promising candidate in AS research. Therefore, innovative strategies by Piezo1 pharmacological approaches will benefit clinical therapy in AS.

### Osteoarthritis

OA is an age-related, chronic and degenerative joint disease characterized by cartilage degradation, bone sclerosis, and persistent inflammation responses in the joints (72). Current knowledge has established that the major cause of OA is the degeneration and degradation of articular cartilage, which is closely related to osteoblasts and osteoclasts (72). To date, several lines of evidence have clearly uncovered the involvement of Piezo1 in cellular processes of osteoblasts and osteoclasts, thus regulating the occurrence and development of OA.

Recently, a study has confirmed the high expression of Piezo1 in bone and skeletal cells (73). More specifically, Piezo1 is expressed in osteocytes, bone marrow mesenchymal stem cells, chondrocytes, osteoblasts, and osteoclasts (74). In osteoblasts, Piezo1 can sense the weight-bearing-induced mechanical stress (73). Knockout of Piezo1 in osteoblast lineage cells disrupts the osteogenesis of osteoblasts and severely impairs bone structure and strength (75). Furthermore, Piezo1 deficiency results in loss of bone mass and spontaneous fractures (73, 75, 76) with enhanced bone resorption by regulating the YAP-dependent expression of type II and IX collagens to increase both the number and activity of osteoclasts (73). There are similar results that loss of Piezo1 leads to multiple spontaneous bone fractures in newborn mice due to inhibition of osteoblast differentiation and increases bone resorption by reducing the formation and activation of NFAT-YAP1-β-catenin (77). Importantly, administration of Yoda1, a Piezo1 agonist, increased bone mass *in vivo* (78). However, with the mechanical loading deleted, Piezo1-deficient mice are resistant to further bone loss and bone resorption (73, 75, 77). As for osteoclasts, Piezo1 is involved in low stimulus amplitude-induced osteoclast differentiation by inducing the release of osteoclast-stimulating factors by hematopoietic progenitor cells (79). Surprisingly, deletion of Piezo1 cannot affect bone resorption and bone mass in mice *in vivo* (73).

The above findings revealed the important role of Piezo1 in bone homeostasis. Nevertheless, the panorama of Piezo1 function in OA remains to be revealed, which is worthy of further exploration.

## Conclusive remarks and perspectives

Mechanotransduction is a process in which mechanical cues are converted into biological signals and has been shown to affect the innate immune cells and multiple disease states. Piezo1 protein has been intensively studied since its discovery as a mechanosensor. In this review, we introduce the structure, activation mechanisms, and pharmacological regulation of the Piezo1 channel. Furthermore, we summarize recent studies on the procession and functions of Piezo1 in macrophages and macrophage-mediated inflammatory diseases. The studies demonstrate that Piezo1 is a vital regulator of macrophage function and polarization in response to various stimuli, such as mechanical stress and inflammatory mediators. However, data on the exact mechanisms underlying the observed phenotypic characteristics are lacking. In addition, many other membrane molecules serve as mechanoreceptors, including integrin, selectin, and cadherin. Whether Piezo1 can interact with these membrane molecules to regulate macrophage function remains to be further studied.

We have also unraveled that Piezo1 is involved in macrophage-mediated inflammatory diseases, such as AD, pulmonary inflammation, atherosclerosis, and OA. The discovery of Piezo1 provides a new insight for the occurrence and development of inflammatory events in the stage after mechanical damage. In addition, the treatment of some clinical diseases requires the implantation of materials, which will produce mechanical force and lead to inflammation and affect the prognosis of the disease. Hence, inflammation and Piezo1 drugs for clinical application will become the focus of future research.

## Author contributions

YT and CZ drafted the original manuscripts. YZ reviewed and edited the manuscript. AZ and MW provided some constructive comments on the structure of the manuscripts. WZ and LZ provided funding. All authors contributed to the article and approved the submitted version.
